# Effect of solvent evaporation on the liquid-crystalline order of itraconazole

**DOI:** 10.1038/s41598-025-23979-9

**Published:** 2025-11-17

**Authors:** Taoufik Lamrani, Luiza Orszulak, Magdalena Tarnacka, Barbara Hachula, Klaudia Nowakowska, Kamil Kaminski, Karolina Jurkiewicz

**Affiliations:** 1 Institute of Physics, Faculty of Science and Technology, University of Silesia in Katowice, 75 Pulku Piechoty 1, 41-500 Chorzow, Poland; 2 Institute of Chemistry, Faculty of Science and Technology, University of Silesia in Katowice, Szkolna 9, 40-006 Katowice, Poland; 3 Department of Biomedical Physics, Faculty of Physics and Astronomy, Adam Mickiewicz University in Poznan, Uniwersytetu Poznanskiego 2, 61-614 Poznan, Poland

**Keywords:** Itraconazole, Solvent-evaporation, Liquid-crystalline structure, Smectic order, Dichloromethane, Applied physics, Chemical physics, Condensed-matter physics

## Abstract

Solvent evaporation is widely used for the formation of amorphous active pharmaceutical ingredients. This study investigates the effect of solvent evaporation on the structure of itraconazole – an antifungal drug and a model thermotropic liquid crystal that exhibits nematic and smectic phases when supercooled from the melt, and when cooled below the glass transition temperature at ~ 330 K, it forms glass with frozen liquid-crystalline order. Using X-ray scattering, it is demonstrated that the solid form of itraconazole obtained at room temperature via evaporation of dichloromethane reveals a smectic order extending up to ~ 15 nm, which is about two times more than what is observed for itraconazole glass obtained by the conventional vitrification. Moreover, the degree of the smectic order may be tuned by adjusting the concentration of the drug in the solution or the choice of solvent. The obtained results are in agreement with previous reports, which indicated an enhancement of smectic order in the system of itraconazole with glycerol derived by evaporation from dichloromethane as compared to pure itraconazole glass [Fatina, C. et al. (2024). *Cryst. Growth & Des.*, 24(11), 4596–4603]. Nevertheless, the new insight has clearly shown that glycerol stabilizing the liquid-crystalline order in itraconazole is not required at all for its generation. This can be achieved with pure itraconazole by evaporation of solvent. Moreover, the presence of glycerol does not extend the long-term room temperature physical stability of the smectic structure in itraconazole. Our findings may be used for producing non-crystalline formulations offering improvements in drug release over the conventional crystalline form.

**Supplementary Information:**

The online version contains supplementary material available at 10.1038/s41598-025-23979-9.

## Introduction

Itraconazole (ITZ), a widely recognized azole antifungal agent, serves as an exemplary model for investigating liquid crystalline (LC) phenomena due to its ability to form distinct LC structures under various thermodynamic conditions and treatments^1^. On the market, pure ITZ is available as a crystalline powder in the polymorphic form I^2^, while the most known pharmaceutical product based on ITZ is Sporanox^®^, which is available as both an oral solution and capsules where a solid dispersion formulation is utilized. Upon melting and subsequent cooling from the isotropic (*I*) liquid phase, ITZ undergoes a transition into a nematic ($\:N$) mesophase at temperature ${T_{I - N}}$ ~ 363 K^3^. Further cooling below ${T_{N - Sm}}$ ~ 347 K induces a transformation from the $\:N$ into a smectic ($Sm$) mesophase^3^. When cooled down below its glass transition temperature ${T_g}$ ~ 330 K, ITZ forms a glassy state that may preserve the molecular order of the $Sm$ mesophase^4^. Importantly, Teerakapibal et al.^4^ demonstrated that $Sm$ layering in ITZ can be frustrated by increasing the cooling rate during vitrification, enabling the formation of glasses with different degrees of the $Sm$-to-$\:N$ order. What is more, for cooling rates exceeding ~ 20 K/s, the $\:N$ to $Sm$ transition may be avoided, resulting in formation of glass with only $\:N$ order (with $Sm$ order parameter = 0). In fact numerous reports have shown that different factors, besides cooling rate, such as deposition rate, precipitation temperature, substrate temperature or mechanical stressing, may tune the LC order of ITZ molecules ^5–8^. Vapor deposition experiments have established that depositing of ITZ at different substrate temperatures results in glasses characterized by wide range of molecular orientations, with some deposition conditions providing high levels of $Sm$ order^5^. Further, it has been shown that shear can change the $\:N$ alignment in this API, which indirectly can affect also the $Sm$ alignment upon cooling down below ${T_{N - Sm}}$, as well as the $Sm$ order trapped in the glass upon further cooling below ${T_g}$^6^. The ITZ stressed in the liquid state exhibited a higher $Sm$ order below ${T_g}$ than its non-stressed counterpart. On the other hand, a recent study performed by our group demonstrated that mechanical stress in the form of vibrational or ball milling can disrupt the crystalline and $Sm$ order of solid ITZ, resulting in particles with $\:N$ organization, where, additionally, the lateral intermolecular order can be tuned by adjusting milling time and frequency^7^. Last but not least, microfluidic anti-solvent precipitation techniques were used to produce ITZ nanoparticles with $Sm$ structures wherein the molecular arrangement can be precisely controlled by the precipitation temperature^8^.

Generally, various solvation approaches have been widely utilized to produce ITZ or its binary mixtures with a vast degree of structural disorder. The interest in this topic stems from the fact that crystalline ITZ exhibits extremely low water solubility (1 ng/ml at pH 7.4), which may account for its low oral bioavailability, while amorphization is one way to improve this important parameter. For instance, Mugheirbi et al. reported the formation of $\:N$ order in ITZ particles produced by spray-drying of the API dissolved in dichloromethane (DCM)^9^. Moreover, they demonstrated unique $\:N$-to-$\:Sm$ switching behavior of ITZ via moisture-triggered activation of molecular mobility below the $\:{T}_{g}$. It is worth to note that spray-drying is a very efficient solvent evaporation-based amorphization method, since it allows for extremely rapid solvent evaporation (SE), leading to the creation of amorphous-like particles. Amponsah-Efah et al. used SE from DCM to prepare mixtures of ITZ with glycerol (GLY). They found that the $\:Sm$-to-$\:N$ phase transition was avoided upon heating when the GLY content was 5 wt% or higher^10^. Complementary studies by Rams-Baron et al. using dielectric spectroscopy revealed that the melt-quenched ITZ:GLY system obtained through DCM evaporation exhibits shorter structural relaxation times compared to the pure ITZ glass, indicating that GLY simultaneously stabilizes and fluidizes the $\:Sm$ ITZ phase^11^. In parallel, recent work by Fatina et al. demonstrated that trace GLY strongly enhances the LC order of vitrified ITZ:GLY system obtained via DCM evaporation compared to its pure vitrified ITZ. They stated that 5 wt% of GLY enhances the $\:Sm$ layering of the ITZ host by reducing the random molecular offsets in each layer and creating more compact layers due to the formation of hydrogen bond cross-links between GLY and ITZ molecules within a monolayer. This was evidenced by the diffraction patterns where the second-order diffraction peak (002) due to the LC ordering was more intense than the first-order reflection (001) while the diffraction pattern of the pure vitrified ITZ is usually characterized by less intense (002) than (001) reflection^12^.

The aim of this report is to show an alternative method for the formation of ITZ solid particles with a more extensive $\:Sm$ order compared to that generated by vitrification − a straightforward solvent evaporation at room temperature without adding any other compound like GLY. The effect of the solvent evaporation process on the phase behaviour of the resulting ITZ solid form, the features of its molecular organization, and related physical stability, thermal properties, and molecular relaxation processes was investigated using X-ray scattering, differential scanning calorimetry, and broadband dielectric spectroscopy. The collected results were compared and discussed with reference to the corresponding data obtained for ITZ glass produced by vitrification, pointing out the differences between these two solid structures with trapped LC order. Moreover, infrared spectroscopy studies were performed in order to verify the possible interactions between the molecules of ITZ and solvent, which would bring closer the mechanism of the formation of the $\:Sm$ order during the solvent evaporation.

## Experimental section

### Materials

Itraconazole (ITZ) with a molecular formula of C_35_H_38_Cl_2_N_8_O_4_, a molar mass of 705.64 g/mol, and a purity of > 98%, was purchased from Angene (CAS Number 84625-61-6) as a 1:1:1:1 racemic mixture of four diastereomers (two enantiomeric pairs), each possessing three chiral canters. Dichloromethane (DCM, CAS Number 75-09-2) and chloroform (CLF, CAS Number 67-66-3), both with a purity ≥ 99%, were purchased from Karpinex. Glycerol (GLY, CAS Number 56-81-5), with a purity of 99.5%, was obtained from Sigma-Aldrich.

### Methods

#### Sample preparation

The vitrified ITZ (V-ITZ), appearing as a milky, glass-like solid, was prepared by melting the crystalline powder at 440 K, followed by quenching of the melt at a cooling rate of ~ 1 K/s down to 295 K (room temperature). Such a procedure is further referred to in the manuscript as “vitrification” while the V-ITZ sample serves as a reference system with a $Sm$ order frozen in a glassy state. Solvent-evaporated ITZ (SE-ITZ) samples, exhibiting a milky appearance with foam-like morphology, were obtained as follows: 20 mg portions of commercial crystalline ITZ were added to: 2, 1, 0.67, and, 0.5 ml of DCM, resulting in ITZ:DCM solutions of different concentrations: 10, 20, 30, and 40 mg/ml, respectively, which were then thoroughly mixed in a cylindrical borosilicate glass vial with a 2 cm diameter and a height of 3 cm to ensure homogeneity. The solutions then underwent solvent evaporation under reduced pressure at a rate of ~ 1 ± 0.02 ml/min using two-stage rotary vane CRVPro 6 pumps connected via a Schlenk line until only a minimal volume of the solvent remained. The evaporation rate was calculated by weighting the solvent and measuring the time required for its evaporation that was observed. To ensure the complete removal of the residual solvent, the samples were subsequently transferred to a vacuum desiccator and subjected to drying at 293 K and 4% RH for 12 h. The solvent-evaporated materials obtained from this process are hereinafter referred to as SE-ITZ X (where X represents the initial ITZ concentration solved in DCM: 10, 20, 30, or 40 mg/ml): SE-ITZ 10, SE-ITZ 20, SE-ITZ 30, and SE-ITZ 40, respectively. The residual DCM content was evaluated using thermogravimetric analysis (TGA), and the results confirmed only trace amounts of solvent (< 0.1 wt%) in the studied materials, as shown in **Fig. SI1** in Supplementary Information. A similar procedure as described above was followed for preparing solvent-evaporated itraconazole with glycerol system SE-ITZ:GLY 20 from 20 mg/ml of solved ITZ:GLY in DCM, incorporating GLY at a concentration of 5% w/w refer to ITZ. Moreover, an alternative solvent was also utilized – CLF, and by applying the same methodology as described above, it was possible to obtain SE-ITZ 20 CLF sample from 20 mg/ml of solved ITZ in CLF. It is worth noting that both compounds, DCM and CLF, were used due to high solubility of ITZ in these solvents (3.27 × 10^− 2^ and 5.04 × 10^− 2^ M/M, respectively)^13^, and their relatively low boiling points (313 K and 334 K, respectively) enabling their fast evaporation at room temperature under reduced pressure. Although DCM and CLF are not common solvents for pharmaceutical production, they allowed us to induce the $Sm$ organization of ITZ molecules by the above-described solvent evaporation process, avoiding crystallization.

#### X-ray scattering

The wide-angle X-ray scattering (WAXS) measurements were conducted using a D/Max Rapid II diffractometer (Rigaku Corporation, Japan) equipped with a rotating Ag anode and accelerated using 60 kV voltage and 200 mA current. The WAXS data are standard X-ray diffraction (XRD) data collected at wide-angle range. A graphite (002) monochromator was employed for the incident radiation. The wavelength of the monochromatic Ag *K*_*α*_ beam was $\lambda$ = 0.5608 Å and the size of the beam on the sample was 0.3 mm. Fresh SE-ITZ samples, prepared after being stored overnight in a vacuum chamber, were packed and sealed in borosilicate glass capillaries and subjected to measurements in transmission mode for one hour. The fresh V-ITZ sample, just after melting and cooling down to 295 K, was also measured using the same settings. The temperature *T* of 295 K was maintained throughout all measurements. X-ray scattering patterns were collected using a 2D curved image plate detector, corrected for background (empty capillary), azimuthally integrated, and converted into 1D functions of the scattering intensity versus the scattering angle (2$\theta$). These were then transformed to the scale of the scattering vector $Q=4\pi \sin \left( \theta \right)/\lambda$, covering a range of approximately 0.17–20 Å^−1^ and normalized with respect to the intensity at the high $\:Q$ range of approximately 4–20 Å^−1^ where the scattering mostly from the ITZ intramolecular structure contributes to the scattering pattern (**Fig. SI2** in Supplementary Information). Our results indicated that the SE-ITZ and V-ITZ samples have the same intramolecular structure, as expected. Therefore, here, the diffraction patterns are presented in only the $\:Q$ range of 0.17–2.65 Å^−1^, where the intermolecular structure primarily contributes. Moreover, the WAXS data for selected ITZ samples were corrected for polarization, absorption, incoherent Compton scattering, normalized to the electron units, and transformed to the structure factor $S\left( Q \right)$ as follows:

$$S\left( Q \right)={\text{~}}\frac{{I\left( Q \right) - \left( {\left\langle {{f^2}} \right\rangle - <f{>^2}} \right)}}{{<f{>^2}}}$$

where: $I\left( Q \right)$ is the coherently scattered intensity, normalized to electron units, $\left\langle {{f^2}} \right\rangle =\mathop \sum \limits_{{i=1}}^{n} {c_i}f_{i}^{2}$, $\left\langle f \right\rangle =\mathop \sum \limits_{{i=1}}^{n} {c_i}{f_i}$, ${c_i}$ and ${f_i}$ are the concentration and the atomic scattering factor of the *i*-th atomic species, respectively, and *n* is the number of atomic species in the sample. The structure factor was then converted by the sine Fourier transform to the real space representation of diffraction data ─ the pair distribution function ($PDF$):

$$PDF\left( r \right)=G\left( r \right)=4\pi r\left[ {\rho \left( r \right) - {\rho _0}} \right]=\frac{2}{\pi }\mathop \smallint \limits_{0}^{{{Q_{max}}}} Q\left[ {S\left( Q \right) - 1} \right]W\left( Q \right)sin\left( {Qr} \right)dQ,$$

where ${Q_{max}}$ is the upper limit of $\:Q$ achieved in the measurements (20 Å^−1^). The window function $W\left( Q \right)=sin\frac{{\left( {\frac{{\pi Q}}{{{Q_{max}}}}} \right)}}{{\left( {\frac{{\pi Q}}{{{Q_{max}}}}} \right)}}$ was used to minimize the termination oscillations. The range of $PDF$’s oscillations was used to quantitatively describe the spatial extent of structural order (the coherence length) in the studied $Sm$ forms. Additionally, small-angle X-ray scattering (SAXS) studies for V-ITZ and SE-ITZ 20 samples were performed using a laboratory Xenocs Xeuss 2.0 SAXS (Xenocs, France) system located at the Joint Laboratory for SAXS studies at the Faculty of Physics and Astronomy, Adam Mickiewicz University. The system was equipped with a MetalJet D2 microfocus X-ray source ($\lambda$ = 1.34 Å) with a liquid metal-jet anode (gallium alloy). SAXS data were collected for powder samples placed between two Kapton films at *T* of 295 K and recorded using a Pilatus3R 1 M hybrid photon counting detector in the $\:Q$ range of approximately 0.05–1 Å^−1^. The distance between the sample and the detector was approximately 580 mm.

#### Differential scanning calorimetry (DSC)

Calorimetric measurements of ITZ were carried out by Mettler-Toledo DSC apparatus (Mettler-Toledo International, Switzerland) equipped with a liquid nitrogen cooling accessory and an HSS8 ceramic sensor (heat flux sensor with 120 thermocouples). Temperature and enthalpy calibrations were performed by using indium and zinc standards. The sample was prepared in an open aluminum crucible (40 µl) outside the DSC apparatus. All samples were measured as followed: at first, they were heated from 325 to 450 K, cooled down to 325 K and then heated again to 450 K at a constant heating/cooling rate of 10 K/min.

#### Broadband dielectric spectroscopy (BDS)

Isobaric measurements of the complex dielectric permittivity ${\varepsilon ^*}\left( \omega \right)$ = $\varepsilon '\left( \omega \right)$ − i$\varepsilon ''\left( \omega \right)$, where $\omega$ is frequency, $\varepsilon '$ and $\varepsilon ''$ represent the real and imaginary parts of ${\varepsilon ^*}$, were carried out for SE-ITZ 20 and V-ITZ samples using a Novocontrol Alpha dielectric spectrometer (Novocontrol Technologies GmbH & Co. KG, Germany) over the $\omega$ range of 10^−1^ – 10^6^ Hz at ambient pressure. The temperature was maintained with a Quatro Cryosystem using a nitrogen gas cryostat with a control better than 0.1 K. Approximately 60 mg of each sample in the powder form was placed between two stainless-steel electrodes, separated by a thin Teflon spacer (diameter of 15 mm, gap of 0.1 mm). The dielectric spectra were investigated in *T* range of 333–385 K.

To obtain relaxation times ($\tau$), the dielectric loss spectra were fitted using the Havrilak-Negami (HN) function^14^ with dc-conductivity (DC) term:

1 $${\varepsilon ^*}\left( \omega \right)=\frac{{{\sigma _{DC}}}}{{{\varepsilon _f}\omega }}+{\varepsilon _\infty }+\mathop \sum \limits_{{i=1}}^{2} \left( {\frac{{\Delta {\varepsilon _i}}}{{{{\left[ {1+{{\left( {i\omega {\tau _{HN,i}}} \right)}^{{\alpha _{HN,i}}}}} \right]}^{{\beta _{HN,i}}}}}}} \right)$$

where ${\sigma _{DC}}$ is the DC factor, ${\varepsilon _f}$ is the permittivity of the free space, $\Delta \varepsilon$ is the dielectric strength, $\omega$ is the angular frequency ($\omega =2\pi f$), ${\tau _{HN}}$ describes HN relaxation time and ${\alpha _{HN}},~{\beta _{HN}}$ are the shape parameters. The subscript *i* is referred to each process. The temperature dependences of the structural relaxation times were fitted using the Vogel-Fulcher-Tamman (VFT) function^15–17^:

2 $$\tau ={\tau _\infty }\exp \left( {\frac{B}{{T - {T_0}}}} \right)$$

where, ${\tau _\infty}$ is the relaxation time in the limit of very high temperatures, ${T_0}$ is the so-called Vogel temperature, and *B* is the activation parameter. ${T_g}$ of all examined samples were determined as the temperature at which structural relaxation time ${\tau _\alpha }$ = 100 s, by extrapolating the VFT fits.

#### Fourier transform infrared (FTIR) spectroscopy

FTIR spectra for ITZ:DCM 20, ITZ:DCM 30, and ITZ:DCM 40 mixtures were measured using a Nicolet iS50 spectrometer (Thermo Fisher Scientific, USA) at a spectral resolution of 4 cm⁻¹ with 16 scans. The spectra were recorded in ATR mode over the wavenumber ($\:\nu\:$) range of 4000–400 cm^− 1^ at 295 K. During the measurements, the FTIR spectrometer was purged with liquid nitrogen to remove water vapour and carbon dioxide from the instrument effectively.

#### Drug release tests

To assess the aqueous release profiles of different ITZ forms, V-ITZ, SE-ITZ 40 and crystalline ITZ, their release kinetics in 0.1 M HCl water solution were measured. The details of the experiment and the registered release curves can be found in the Supplementary Information.

## Results and discussion

### Impact of evaporation rate on the resulting structure of solvent-evaporated itraconazole

**Fig. 1:**
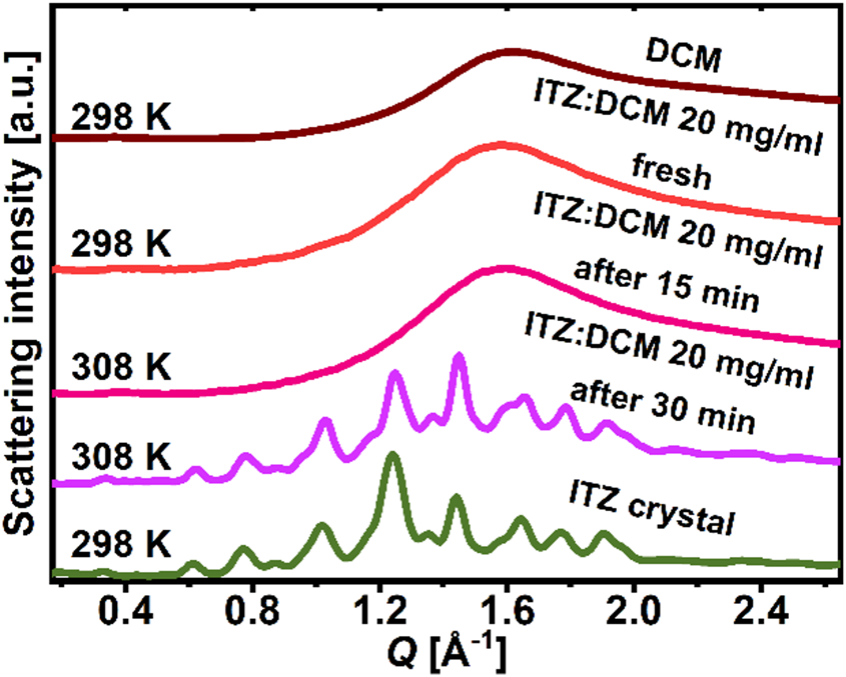
XRD patterns of freshly prepared ITZ:DCM solution of 20 mg/ml, measured at 295 K, and the solution after 15 and 30 min at 308 K, compared with the patterns for pure crystalline ITZ and liquid DCM at 295 K.

Initially, in order to assess the structural behavior of the ITZ:DCM solution during DCM evaporation, the XRD experiment was conducted for the solution sealed in a glass capillary. As shown in **Fig. ****1**, the XRD pattern of freshly prepared ITZ:DCM mixture of 20 mg/ml exhibited only one broad halo at *Q* ~ 1.6 Å^−1^, with no evidence of LC or crystalline ordering, suggesting that ITZ was fully dispersed in DCM. The sample was subsequently heated from 295 up to 308 K to facilitate faster DCM evaporation. Notably, after 30 min, when DCM evaporated, the residual ITZ crystallized to the same polymorphic form (I) as the commercial reference ITZ. A number of similar experiments were performed for ITZ:DCM solutions with various ITZ concentrations. However, the resulting structure of ITZ obtained by these experiments was always crystalline. The solvent evaporation rate in these experiments was much slower (~ 0.01 ± 0.005 ml/min) than in the case of the evaporation experiments performed under reduced pressure (~ 1 ± 0.02 ml/min), as described in the Sample preparation section. We refer to these two evaporation rates below as “slower” and “faster”. The crystallization of ITZ occurred in less than 30 min, and the time resolution of the XRD measurements did not allow observing whether any intermediate LC phases were formed before crystallization.

**Fig. 2:**
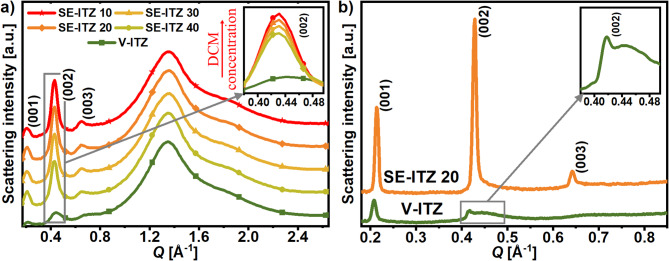
(a) XRD patterns of V-ITZ and SE-ITZ samples prepared from ITZ:DCM mixtures of different concentrations (10, 20, 30, and 40 mg/ml). A comparison of the (002) reflection between SE-ITZ samples and V-ITZ is given as an inset to panel (a). (b) SAXS patterns of V-ITZ and SE-ITZ 20. The inset to panel (b) provides a zoomed-in view on the (002) peak for V-ITZ.

Unfortunately, using this XRD setup it was not possible to track the structural changes in-situ during the solvent evaporation process under reduced pressure, which was used to prepare target SE-ITZ samples, as described in the Sample preparation section. Therefore, the structure of SE-ITZ samples was studied ex-situ after complete DCM removal. Their diffraction patterns, presented in **Fig.** **2**, indicate that they are not characterized by either crystalline or amorphous structure but exhibit a $\:Sm$ order manifested by the presence of (001), (002), and (003) reflections due to the layered organization. These findings underscore the influence of the solvent evaporation rate on the resulting organization of molecules in ITZ, as the slower DCM evaporation favored ITZ crystallization while the faster solvent removal resulted in the formation of $\:Sm$ order. In contrast, it is worth to remind that ITZ particles obtained by spray drying, which involves extremely rapid solvent removal, exhibited a lower degree of structural order (namely, nematic order in this case)^9^ than the SE-ITZ samples studied here. Thus, it seems that using different solvent evaporation rate scales, ITZ can be obtained in solid crystalline, $\:Sm$, or $\:N$ forms, while the higher the evaporation rate, the higher the resulting structural disorder of ITZ molecules.

### Liquid crystalline order of the solvent-evaporated itraconazole

Here, it is presented that the DCM evaporation from the ITZ:DCM solution may lead to the formation of a LC order in ITZ, instead of its crystallization, when a faster evaporation rate (1 ml/min) is applied than that reported for XRD experiment in closed capillaries (0.01 ml/min), as described in the Sample preparation section. The diffraction patterns of such prepared SE-ITZ samples after complete DCM removal are presented in **Fig.** **2****a**. The XRD curves for all these samples exhibit a broad main peak at $\:Q$ ~ 1.35 Å^−1^, corresponding to the lateral packing of ITZ molecules. The main peak’s position indicates an average intermolecular distance $d=2\pi /Q$ ∼ 4.65 Å. Additional peaks in the low-*Q* region suggest the presence of LC order. Specifically, for ITZ molecules arranged in $Sm$ layers, a series of diffraction peaks appear at $Q~\sim {\text{~}}$0.21, 0.43, and 0.65 Å^−1^, consistent with previously reported patterns in the literature^4,18^. These peaks indicate a LC periodic structure with a periodicity $d~\sim {\text{~}}$30 Å^19^, which is approximately the length of ITZ molecule^20^. Due to a limitation of the setup used for the WAXS measurements, the intensity of the first-order diffraction peak (001) from the layer organization of ITZ molecules is partially obscured by the beam stopper and cannot be taken into analysis. Additionally, the third peak, observed at$~Q$ ~ 0.65 Å^−1^, is notably weaker. Therefore, the second-order diffraction overtone (002) at *Q* ~ 0.43 Å^−1^ (inset in **Fig. ****2****a**) was analyzed to assess the features of the LC order in the SE-ITZ samples. Just to note, all diffraction data presented in **Fig. ****2****a** were scaled to the same background level for high $\:Q$-values (4–20 Å^−1^), which depend mainly on the intramolecular structure, so one can compare the relative peak amplitudes at low $\:Q$values (< 4 Å^−1^) arising mainly due to intermolecular structure. One can see that there is a clear trend in the (002) peak’s amplitude as a function of ITZ concentration – it is the highest for ITZ derived from the most diluted solution in DCM (10 mg/ml) and subsequently decreases with the higher concentration of ITZ. Thus, this finding demonstrates that the degree of the $Sm$ order of SE-ITZ can be tuned by adjusting the ITZ:DCM concentration in the solution prior to solvent evaporation. Generally, the greater the amplitude of the diffraction peaks, the smaller their full width at half maximum (FWHM), which indicates the increase in the correlation length within the $Sm$ layering. A similar trend was also observed for the third-order (003) peak. On the other hand, the positions of the (002), (003) and the main diffraction peaks for SE-ITZ samples do not vary with ITZ concentration. This shows that the nature of the $Sm$ as well as the lateral packing, including the molecular spacings between and within $Sm$ layers in the SE-ITZ solid particles, is essentially the same regardless of ITZ concentration in the initial ITZ:DCM solution.

In order to gain deeper knowledge about the nature of the $Sm$ order in the SE-ITZ materials, their diffraction patterns were then compared with the pattern of ITZ glass obtained by vitrification (V-ITZ), presented also in **Fig.** **2****a**. The (002) reflection for V-ITZ is much more damped and broader than those registered for SE-ITZs. What is more, the position of the (002) peak’s maximum for V-ITZ is clearly shifted towards higher $\:Q$~ 0.45 Å^−1^ compared to that registered for SE-ITZs: $\:Q$ ~ 0.43 Å^−1^. Such $\:Q$ positions correspond to interlayer distances $d~\sim$ 28 and 29 Å, respectively. So, the average distance between $Sm$ layers in V-ITZ is shorter than that of SE-ITZ. A smaller spacing between $Sm$ layers can be due to a tilt of molecules in the layers from $Sm~A$ towards $Sm~C$ arrangement or due to a reduction of offsets of molecules in the direction perpendicular to layers, which promote a disorder in the layer-like organization. More insight into the organization of the ITZ molecules in the studied V-ITZ and SE-ITZ forms was obtained from the SAXS patterns collected for lower $\:Q$-values than the WAXS data, and allowed us to register (002), (003) as well as the (001) reflections due to the layered organization.

Figure 2b presents the comparison between the SAXS curves of SE-ITZ 20 and V-ITZ. The SAXS data differ from WAXS patterns presented in Fig. 2a in terms of the probed $\:Q$ range and resolution. However, the most important finding revealed by WAXS diffraction patterns that SE-ITZ samples show much more intense and sharper (002) and (003) peaks than V-ITZ is also confirmed by the SAXS patterns. Nevertheless, the SAXS patterns provide some interesting features about the $Sm$ structures of these two ITZ forms. First of all, the (002) peak of V-ITZ is comprised of two components: a more sharp feature at $\:Q$ ~ 0.42 Å^−1^ and a broader bump at $\:Q$ ~ 0.44 Å^−1^. In WAXS pattern, these two contributions merge in one peak at $\:Q$ ~ 0.43 Å^−1^. A similar two-component feature of the second-order reflection from ITZ layers was found by Fatina et al. and Yu et al. for ITZ glass prepared by melt-quenching^12,21^. The sharper component was attributed to the $Sm$ layering while the broader one to the excluded volume effect^21,22^. The first order (001) peak for V-ITZ on the SAXS curve has a higher intensity than the (002) one. A similar behavior was also found in previous studies of ITZ glasses^12,21^. In contrast, for SE-ITZ 20, one can see that the (002) peak on the SAXS curve is stronger than the (001) one, which deviates from the typical diffraction pattern for LC structures. Fatina et al. proposed a structural model to quantitatively explain such an effect. They showed that the relative intensities of (001) and (002) reflections in ITZ contain information on the density modulation perpendicular to the$~Sm$ layers and depend strongly on the intralayer molecular offset. A reduction of the random molecular offsets results in an increase in the intensity of the (002) peak compared to the (001) peak, and for the perfect arrangement of ITZ molecules with no offset in the direction perpendicular to the layers, the ratio of the (002)/(001) peaks’ intensities is around 2. They observed that such a situation occurs in ITZ:GLY system obtained through DCM evaporation and proposed that no offset ordering of ITZ molecules in $Sm$ layers is due to GLY cross-linking of ITZ by hydrogen bonds within a monolayer. Here, we reveal that a similar $Sm$ structure to that reported by Fatina et al. for ITZ system containing 5% w/w of GLY may be achieved by evaporation of ITZ from the DCM solvent without the participation of GLY. Therefore, the formation of the ITZ $Sm$ order with the reduced intralayer molecular offset seems to be the characteristic feature of the used SE method itself, not the effect of GLY. The correctness of the hypothesis about the reduced offsets of molecules in the SE-derived ITZ is supported by the behaviour of positions of the diffraction peaks in the SAXS pattern. For the SE-ITZ sample, the peaks are clearly shifted towards higher $\:Q$-values, compared to peaks for V-ITZ, indicating lower interlayer spacing in the SE-ITZ than in the ITZ glass.

**Fig. 3:**
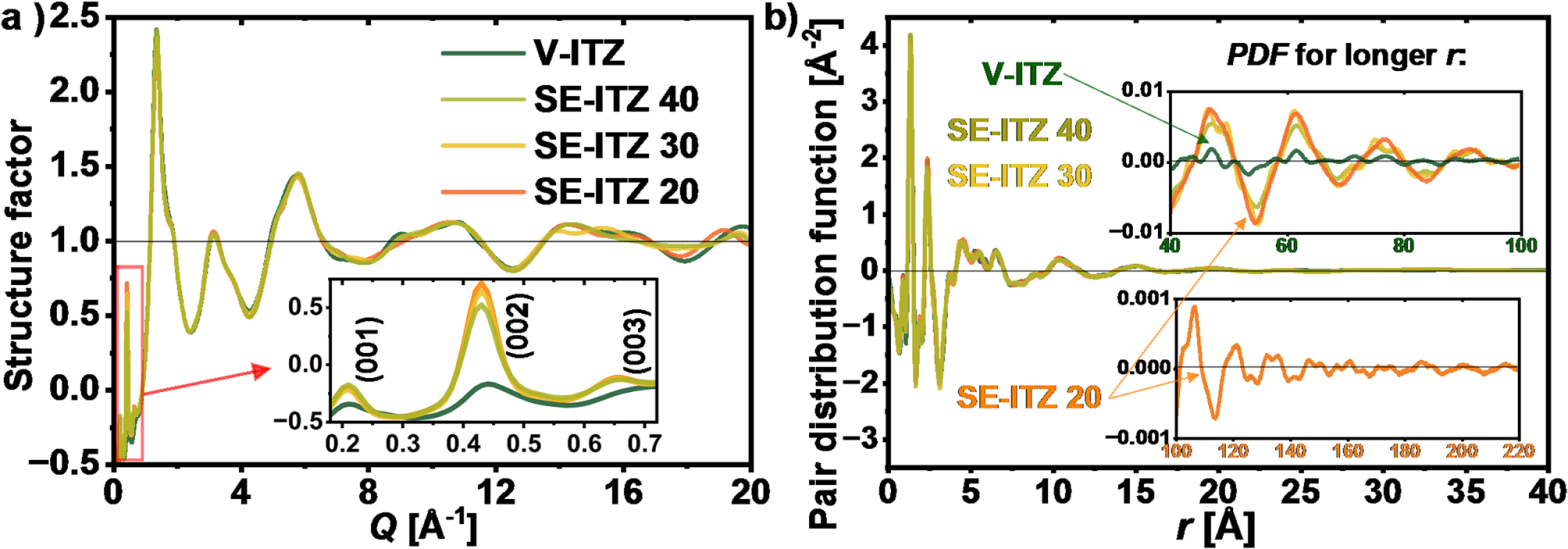
(a) Structure factors and (b) pair distribution functions derived from WAXS data for V-ITZ and selected SE-ITZ samples. The insets in panel b) show the extent of structural correlations where $\:PDF$ oscillations gone, which is ~ 70 Å for V-ITZ and ~ 150 Å for SE-ITZ 20.

In order to characterize the $Sm$ structures of SE-ITZs and V-ITZ quantitatively, the WAXS data were normalized to electron units and presented as structure factors in **Fig. ****3****a**. As can be seen, the trend in the (002) peak’s amplitude revealed in corresponding diffraction patterns in **Fig. ****2****a** is also maintained for the structure factor representation of the diffraction data. The ratio of the (002) peak’s amplitude in the $S\left( Q \right)$ data for V-ITZ and SE-ITZ 20 is ∼ 1:3. Further, to quantify the coherence length of the atomic correlations in the $Sm$ structures of SE-ITZs and V-ITZ, the $S\left( Q \right)$ data were transformed to the atomic pair distribution functions, as described in the Methods section, and the $PDF$s were presented in** Fig. ****3****b**. As can be seen, the first peaks of the $PDF$s for small distances, representing the intramolecular and short-range intermolecular correlations, almost overlap for V-ITZ and SE-ITZs. However, there is a clear difference in the extent of the $PDF$’s oscillations between V-ITZ and SE-ITZs. For the V-ITZ system, the structural correlations are completely attenuated at ~ 70 Å. On the other hand, the $PDF$’s oscillations for the SE-ITZ systems extend to about 150 Å, indicating longer-range ordering of these forms as compared to V-ITZ. Moreover, the differences in the coherence lengths between the different SE-ITZ samples were very small, reaching a few Å between SE-ITZ 20 and SE-ITZ 40, where the latter one exhibited the faster decay of the PDF’s oscillations, as visible in the upper inset to **Fig. ****3****b**.

The X-ray scattering data presented above were collected for fresh SE-ITZ samples – up to 2 days after the solvent evaporation procedure. Further, in order to verify whether the $\:Sm$ order generated by the SE method is stable, the long-term physical stability of these materials was monitored, which is especially important for potential applications of APIs in pharmacy.

### Physical stability of the solvent-evaporated itraconazole materials below the glass transition temperature

**Fig. 4:**
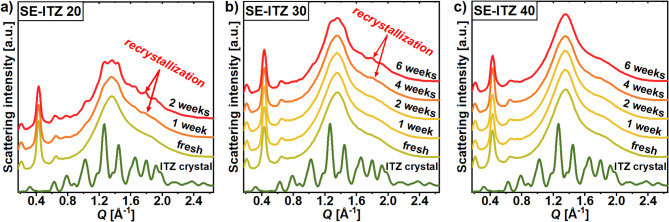
Temporal evolution of XRD patterns for SE-ITZ samples prepared from different ITZ:DCM concentrations: (a) 20 mg/ml, (b) 30 mg/ml, and (c) 40 mg/ml, at room conditions.

Figure 4 presents the temporal evolution of the WAXS patterns recorded weekly for SE-ITZ samples stored under room conditions (*T* ~ 295 K, ambient pressure and humidity) in sealed capillaries. It is worth mentioning that in one of the recent papers by our group^7^, it was demonstrated that V-ITZ may be stable in its $Sm$ form in such conditions for up to approximately 5 weeks while ITZ samples generated by cryo-milling and exhibiting a lower structural order (*N*) than V-ITZ were better physically-stable and did not crystallize for at least 10 weeks when stored at analogous conditions. What is more, it was presented that the resistance to the crystallization of the cryo-milled ITZ samples increases with a lower degree of the short-range order (quantified by the main diffraction peak amplitude and FWHM) whereas the $\:N$ order was comparable between these samples. Therefore, the diffraction patterns of the SE-ITZ samples with different coherence lengths of the $Sm$ order were probed over time to explore the relationship between the features of their $Sm$ structures and physical stability. As can be deduced from the results presented in **Fig. ****4**, there is a trend between the amplitude of the second-order (002) diffraction peak for the fresh SE-ITZ samples and their physical stability. Namely, the lower the amplitude, the better the stability. Over time, the amplitude of the (002) peak minimally decreased for all SE samples. Then, SE-ITZ 20 underwent crystallization after just 1 week, SE-ITZ 30 showed signs of crystallization after 4 weeks, while SE-ITZ 40 revealed the best stability, among the studied samples, for at least 6 weeks. Additional diffraction measurements performed on SE-ITZ 40 sample stored 5 months still did not reveal crystallization, as presented in **Fig. SI3**, so SE-ITZ 40 material is characterized by at least two times longer physical stability than SE-ITZ 30 and V-ITZ, in the same conditions.

Due to the promising, from the pharmaceutical industry point of view, physical stability of the SE-ITZ 40, in order to further explore the potential of this ITZ form for pharmaceutical applications, we decided to test its drug release kinetics in 0.1 M HCl aqueous solution, simulating the acidic conditions of the stomach, and compare this solubility with that of crystalline as well as glass ITZ forms. It is widely accepted that fasting gastric emptying usually takes about 2 h^23,24^. Therefore, in our study, we adopted a measurement duration of approximately 3 h. The registered drug released profiles are included in Supplementary Information as **Fig.** SI7, and they show that the amount of released of SE-ITZ 40 after 3 h reached ~ 0.037 mg/ml while that of crystalline ITZ was around 7 x lower (~ 0.005 mg/ml). Herein, it should be noted that the solubility of crystalline ITZ in an acidic environment is ~ 0.004 mg/ml at 297 K^25^. In turn, our studies conducted at a temperature reflecting the human body (310 K) showed a slightly higher amount of dissolved and released active substance (~ 0.005 mg/ml), which is solely due to the temperature effect, and the amount of released API corresponds well with literature data. Moreover, the concentration of released of V-ITZ after 3 h was slightly lower (~ 0.034 mg/ml) than that of SE-ITZ 40. Thus, both non-crystalline, SE-ITZ and V-ITZ, show much better bioavailability than crystalline ITZ in 0.1 M HCl. However, the differences in release profiles due to the differences in the degree and length of the $Sm$ order between SE-ITZ and V-ITZ are negligible or may be blurred due to their distinct morphology, which is known to highly affect the solubility as well.

Additionally, to examine the influence of an elevated temperature, but still below ${T_g}$, on the SE-ITZ physical stability, WAXS measurements were conducted for SE-ITZ 30 stored at *T* = 313 K, ambient pressure and humidity. Just to remind, at room temperature, SE-ITZ 30 showed a similar stability as V-ITZ (approximately 4–5 weeks). As shown in **Fig. SI4** in Supplementary Information, the amplitude of the second-order (002) diffraction peak of the SE-ITZ 30 at 313 K was gradually decreasing over the storage time of 14 h, trending toward the amplitude of the V-ITZ. However, the crystallization of the SE-ITZ 30 at 313 K was registered after 15 h, before the amplitude of its (002) peak merged with the amplitude of the V-ITZ. Therefore, there is no trend in the amplitude of the LC peaks and the physical stability of ITZ species produced by the SE method and vitrification, which may imply a different characteristic of their $Sm$ order and related properties such as the molecular dynamics, which will be discussed further.

### The effect of glycerol on the liquid-crystalline order of the solvent-evaporated itraconazole

**Fig. 5:**
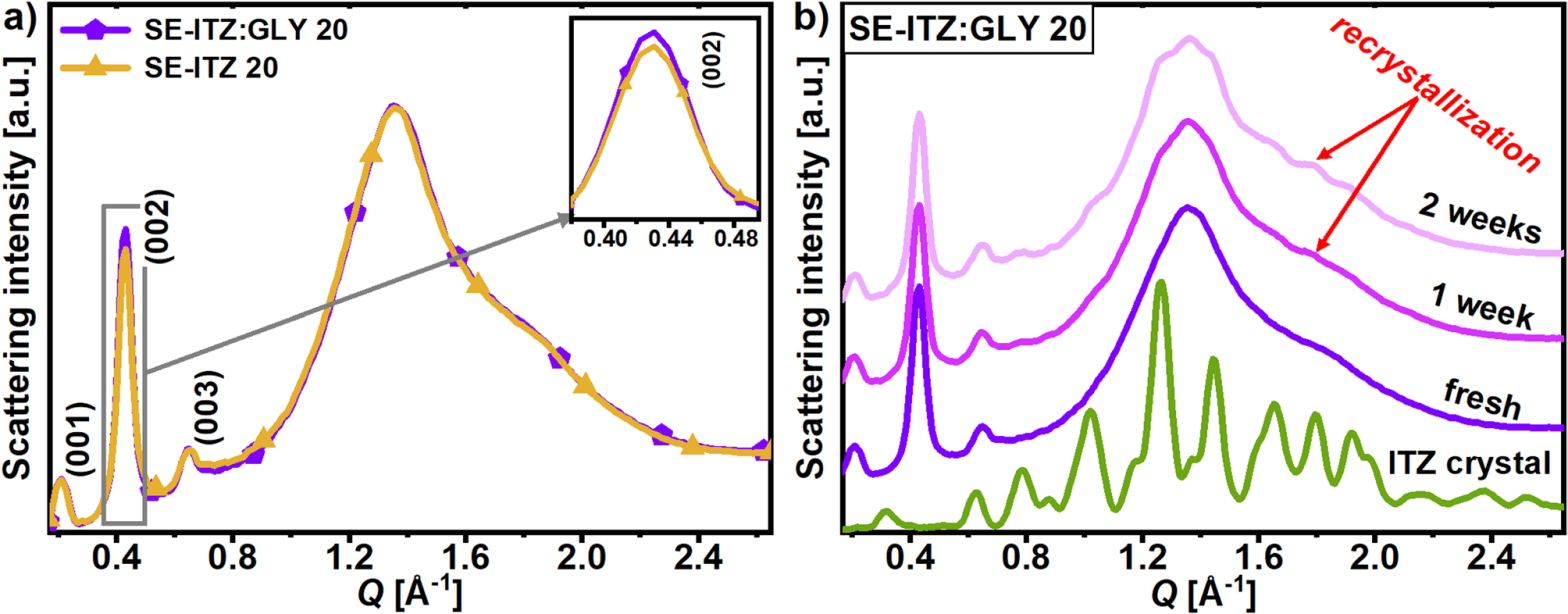
(a) Comparison of XRD patterns for fresh SE-ITZ 20 and SE-ITZ:GLY 20 samples. The inset in panel (a) shows a zoom on the second-order (002) peak. (b) Temporal evolution of the XRD pattern for the SE-ITZ:GLY 20 at room temperature.

As discussed in the Introduction, Fatina et al. demonstrated that GLY enhances the LC order of ITZ:GLY obtained via SE from DCM and vitrified as compared to pure vitrified ITZ^12^. In order to elucidate further the effect of the presence of GLY in the ITZ:DCM mixture on the final LC order of ITZ obtained after DCM evaporation, the SE-ITZ:GLY 20 sample (where 20 indicates 20 mg/ml concentration of ITZ:GLY system in DCM and GLY concentration in ITZ:GLY system was 5% w/w) was prepared, as described in the Sample preparation section, and probed by XRD. The XRD data for this system were compared with the data obtained for SE-ITZ 20 sample. Position, shape and amplitude of the main diffraction peaks for these two freshly prepared systems were very similar, as can be seen in **Fig. ****5****a**. Nonetheless, the SE-ITZ:GLY 20 exhibited a slightly higher intensity of the (002) reflection than the SE-ITZ 20 (inset in **Fig. ****5****a**), beyond measurement uncertainty. This suggests that, indeed, GLY enhances the LC ordering in ITZ during the solvent evaporation.

Nevertheless, as demonstrated in **Fig. ****2****a**, a similar increase in the (002) peak’s amplitude may be induced without involving GLY, by only reducing the ratio of ITZ to DCM in the solution from 20 down to 10 mg/ml. What is more, the XRD studies of the physical stability of the SE-ITZ:GLY 20 exhibited its very similar temporal stability to SE-ITZ 20, as shown in **Fig. ****5****b**. The crystallization of ITZ from the SE-ITZ:GLY 20 system started after one week from the preparation, i.e., after the same period as the SE-ITZ 20 sample, according to the XRD results presented in **Fig. ****4****a**. This suggests that incorporating GLY into the SE-ITZ structure does not improve its resistance to crystallization at room conditions, below the glass transition temperature.

### Impact of solvent type on the structure of the solvent-evaporated itraconazole

**Fig. 6:**
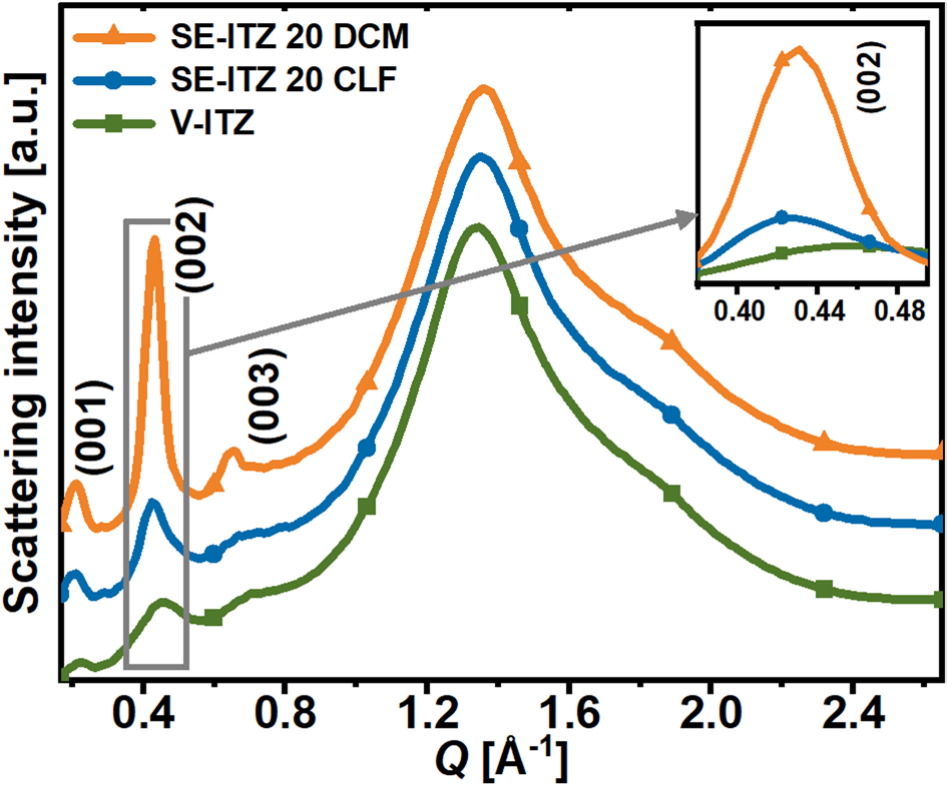
XRD patterns of fresh SE-ITZ 20 samples prepared by solvent evaporation method using DCM (SE-ITZ 20 DCM) and CLF (SE-ITZ 20 CLF) as solvents. The patterns were compared to the data collected for vitrified ITZ (V-ITZ).

To gain further insight into the mechanism of the formation of LC order in ITZ by the solvent evaporation process, an alternative solvent – CLF was employed. CLF was chosen, despite the fact that it is increasingly discouraged by the industry, the same as DCM^26^. However, CLF exhibits the highest ITZ mole fraction solubility at room temperature, among the other solvents, of ~ 5.04 × 10^− 2^, which is 1.54 times higher than that of DCM^13^, while it is difficult to find other solvents for ITZ that ensure its high solubility while having a relatively low boiling point, ensuring rapid evaporation of the solvent at around room temperature. Following the same preparation method of the SE-ITZ 20 system from the solution with DCM, SE-ITZ 20 CLF analog was prepared from the ITZ mixture with CLF, in the same ratio of 20 mg of ITZ per 1 ml of solvent. The XRD pattern in **Fig. ****6** shows that SE-ITZ 20 CLF exhibits much lower amplitudes of the diffraction peaks originating from the $Sm$ order than SE-ITZ 20 DCM, but still, they are more intense than for the V-ITZ system. This suggests that DCM is not the only solvent capable of inducing an enhanced $Sm$ ordering of ITZ, as compared to the ITZ glass obtained by vitrification. However, the question arises as to why DCM supports the formation of a much higher degree of the $Sm$ organization in ITZ than CLF. In this context, other solvents, such as tetrahydrofuran, acetonitrile, acetone, and methanol, were also tested. However, due to lower ITZ solubility by these solvents compared to the chlorinated ones, such as DCM and CLF^13^, the resulting SE-ITZ systems exhibited a crystalline structure, regardless of the ITZ:DCM concentration used. Thus, a high solubility of ITZ in a solvent is one of the most crucial factors for the effective amorphization of ITZ, but its final structure and the degree of the LC order/its coherence length generated by the SE method may depend on other properties of the solvent, such as boiling point, external conditions or interactions between ITZ and solvent, which will be discussed further.

### Thermal transitions of the solvent-evaporated itraconazole systems

**Fig. 7:**
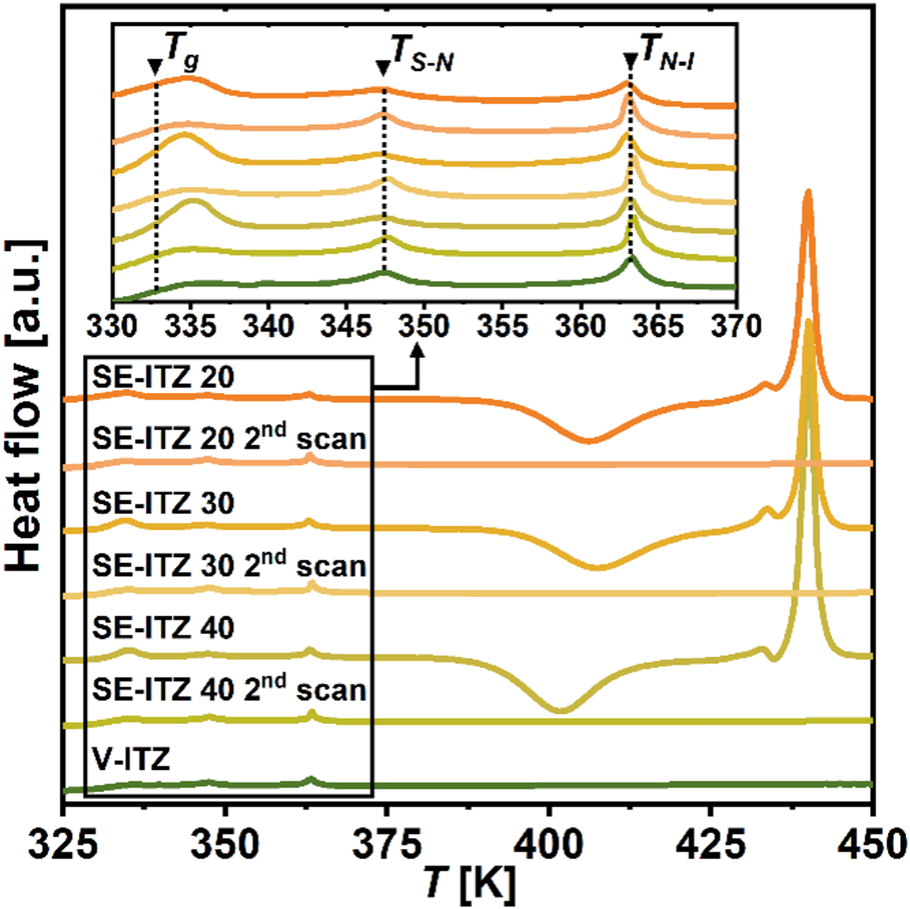
DSC thermograms of SE-ITZ and V-ITZ samples recorded during heating at 10 K/min. The second scans performed on the SE-ITZ samples first heated up to 450 K and cooled down are also presented. The inset shows a zoomed range of the data in the region where the LC transitions occur.

To further investigate the thermal properties of the SE-ITZ systems, calorimetric measurements were performed. The collected thermograms for freshly prepared systems after DCM evaporation and their vitrified derivatives (2nd scans) are compared in **Fig. ****7** with data for V-ITZ. As illustrated, V-ITZ and all SE-ITZ samples exhibit the glass transition, marked at the ${T_g}$ by a heat capacity jump, and two distinct endothermic peaks at ${T_{Sm - N}}~$~ 345 K and ${T_{N - I}}$ ~ 362 K, consistent with the well-known LC behavior of ITZ^3,27^^,^^28^. So, the SE-ITZ systems preserve the sequence of LC transitions reported for the ITZ glass. However, the SE-ITZ materials undergo crystallization above 400 K, followed by melting at ${T_m}~$~ 439 K, in contrast to V-ITZ. One can also notice that SE-ITZ materials show a slight shift in ${T_g}$ (Δ${T_g}$ ~ −3 K) compared to V-ITZ, which may be due to the structural differences between these specimens, i.e., in the degree of the $Sm$ ordering below ${T_g}$, in the size of the $Sm$ domains, and/or in the lateral packing of molecules. However, this effect is fully reversible after melting, as all 2nd DSC scans (obtained after heating SE-ITZ above its melting point and vitrification) matched that of V-ITZ in terms of the temperatures as well as areas under the thermal events. This confirms that the extensive $Sm$ order generated in the SE-ITZ systems is fully disrupted upon melting.

Herein, it is also worth to emphasize that in the thermograms registered for SE samples, one can easily detect enthalpy recovery peak (overshoot) at the $\:{T}_{g}$. Such a feature is observed in the aged glasses. Hence, it might be an indicator of the higher thermodynamic stability of the obtained materials and a higher order of the molecular arrangement in ITZ obtained via solvent evaporation as compared to the vitrified form. A similar finding was also reported for the cryo-milled ITZ^7^.

### Differences in the molecular dynamics of the solvent-evaporated and vitrified itraconazole

**Fig. 8:**
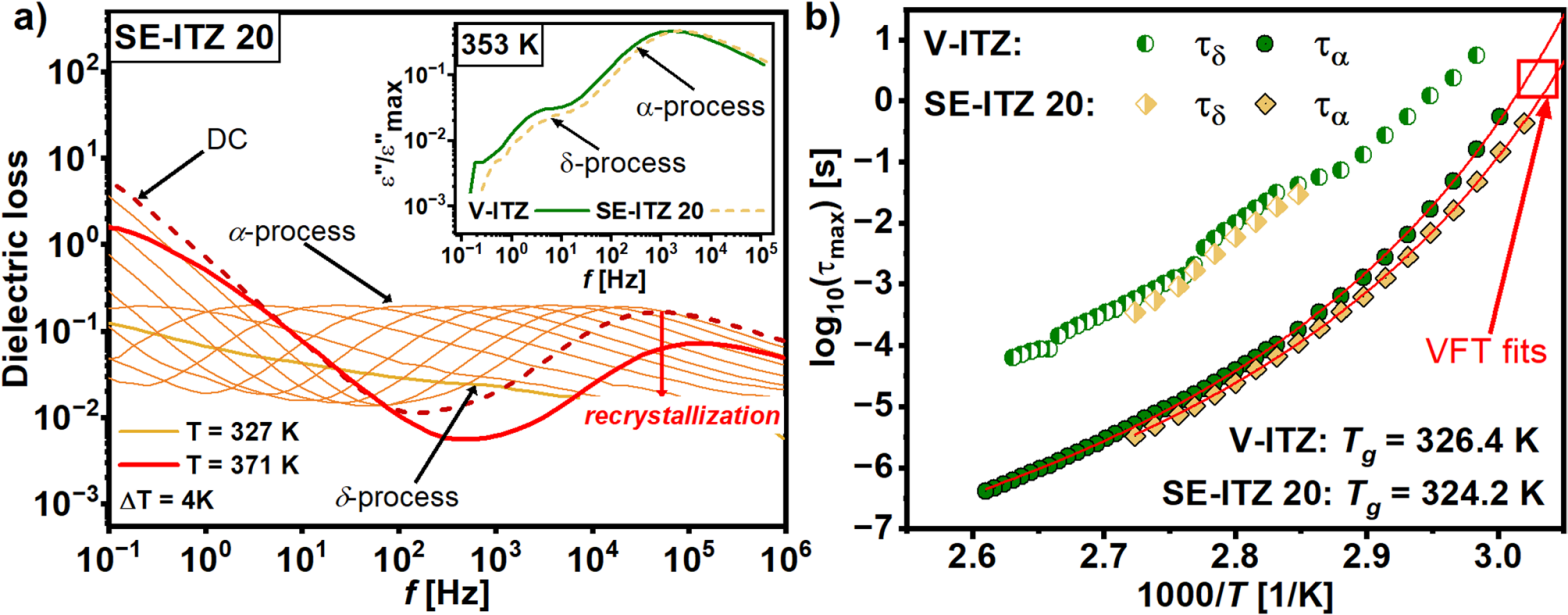
(a) Dielectric loss spectra recorded for SE-ITZ 20 above ${T_g}$. As the inset in panel (a), the comparison of the position of the loss peaks obtained for V-ITZ and SE-ITZ is presented. (b) Relaxation maps of the SE-ITZ and V-ITZ samples.

Moreover, the molecular dynamics of V-ITZ and SE-ITZ systems were investigated by dielectric spectroscopy to verify how the $Sm$ order induced by the SE method influences the relaxation processes of ITZ compared to its glass form obtained by vitrification. Figure 8a shows dielectric loss spectra obtained for SE-ITZ 20, which are composed of three relaxation processes: (*i*) a DC-process (related to the ion transport) at lower frequencies, (*ii*) a prominent structural *α*-relaxation process (reflecting rotations around the long molecular axis) at higher frequencies, that dominates the recorded spectra, and (*iii*) a $\delta$-process (corresponding to molecular reorientation around the short axis), appearing at the ride side of the structural relaxation. As can be seen, upon heating, the amplitude of the *α*-relaxation peak decreases rapidly at higher temperatures (*T* > 360 K), indicating the crystallization process, which agrees with the outcomes of the calorimetric data for SE-ITZ, where crystallization was also observed (**Fig. ****7**). Further, the temperature dependences of the relaxation times $\tau$for the observed relaxation processes were determined, where ${\tau _\alpha }$ and ${\tau _\delta }$ refer to the relaxation times of the $\alpha$ and $\delta$ processes, respectively. The collected dielectric data were fitted using two Havrilak-Negami (HN) functions with the additional DC term^29^ (see Methods). The calculated $\tau \left( T \right)$ dependencies are displayed in **Fig.** **8****b**. As one can see, there are significant differences in the structural dynamics between the V-ITZ and SE-ITZ near ${T_g}$. The SE-ITZ 20 sample exhibits shorter ${\tau _\alpha }$ values compared to their vitrified counterpart. These differences diminish at higher temperatures ~ 363 K, upon approaching the *N*-*I* transition. However, even though the ${\tau _\alpha }$ of the SE-ITZ converges to that of the V-ITZ, it never reaches it. Moreover, there is also a difference in the ${T_g}$ of the V-ITZ and SE-ITZ systems derived from the dielectric data using the Vogel-Fulcher-Tamman (VFT) function^15–17^ (see Methods). Namely, ${T_g}$ ~ 326.4 and 324.2 K for V-ITZ and SE-ITZ 20, respectively, which aligns well with the outcomes of the calorimetric data. Note that ${T_g}$ was defined as *T* where ${\tau _\alpha }$ = 100 s.

Additionally, the $\delta$-process was analyzed at high temperatures (see **Fig. ****8****a**). As one can see, at lower temperatures, SE-ITZ 20 shows slightly faster $\delta$-mode dynamics compared to the V-ITZ, though this difference falls within the experimental uncertainty and disappears at elevated temperatures. Thus, it is evident that the molecular dynamics of both relaxation processes, $\alpha$ and $\delta$, for the SE-ITZ becomes nearly identical to that of its vitrified form at higher temperatures, where ITZ transforms to the liquid phase. The loss of structural differences in the liquid state for ITZ specimens obtained by the distinct methods transfers to the unification of their molecular dynamics. In turn, our results clearly demonstrate that the differences in the LC molecular arrangement in those ITZ forms at lower temperatures yield difference in their molecular dynamics.

### Intermolecular interactions of itraconazole with dichloromethane and discussion on the possible mechanism of the formation of the smectic order in the solvent-evaporated itraconazole

Seeking an explanation of the mechanism behind the formation of the superior $\:Sm$ order in the DCM-evaporated ITZ, as compared to V-ITZ reference, FTIR spectroscopy studies were performed. More precisely, the aim of the FTIR analysis was to verify whether there is any interaction between the solvent and ITZ molecules in the ITZ:DCM mixtures. The spectra collected at 295 K for pure DCM, V-ITZ as well as fresh ITZ:DCM mixtures (with concentrations of 20, 30, and 40 mg/ml) are presented in **Fig. SI8** in Supplementary Information. Itraconazole contains several functional groups capable of interacting with the solvent, including proton donor C–H groups (3100–2700 cm^− 1^), as well as proton acceptor groups such as C = O moieties (around 1700 cm^− 1^), aromatic C = C groups (1600–1500 cm^− 1^), triazole C = N groups (1450–1330 cm^− 1^), dioxolane C–O–C groups (1100–1050 cm⁻¹) and aromatic C-Cl groups (around 1100 cm^− 1^)^30,31^. Thus, the shifts in these characteristic bands can provide insight into which functional groups are engaged in interactions with DCM. Analyzing the spectra illustrated in **Fig. SI8**, it can be observed that most of the bands characteristic of ITZ in the ITZ:DCM mixtures exhibit only minor shifts (by approximately 1–2 cm^− 1^) compared to those in pure vitrified ITZ. The peaks that shift the most under the influence of solvent (by max. 6 cm^− 1^) are those of the aliphatic CH (2936 –2827 cm^− 1^) and the C = O stretching (1750 –1650 cm^− 1^) vibrations. In more detail, the ITZ signal located at 2936 cm^− 1^ after the introduction of solvent (for the ITZ:DCM of 20 mg/ml concentration) is blue-shifted to 2941 cm^− 1^, and the addition of more ITZ to reach higher concentrations leads to the red-shift of this band (to 2938 and 2939 cm^− 1^ for the mixtures of 30 and 40 mg/ml concentrations, respectively). An analogous effect is detected for the band appearing at 2878 cm^− 1^ for V-ITZ. It is shifted to 2884, 2883, and 2882 cm^− 1^ for the ITZ:DCM mixtures of 20, 30, and 40 mg/ml, respectively. In the case of the peak located at 2827 cm^− 1^ for V-ITZ, it is blue-shifted for 20 mg/ml solution as compared to V-ITZ, and then the blue-shift gets more significant with the higher ITZ concentration. These detected C-H band shifts are probably related to weak hydrogen bonding interactions between ITZ and DCM molecules. Moreover, the C = O band of ITZ is blue-shifted from 1697 cm^− 1^ for pure V-ITZ to 1702 cm^− 1^ for ITZ:DCM mixtures with the high ITZ concentration, which can suggest van der Waals interactions between ITZ and DCM molecules. It should also be noted that the IR peak positions originating from DCM also exhibit insignificant shifts (by approximately 1–2 cm^− 1^) in the ITZ:DCM mixtures, compared to those in neat DCM (**Fig. SI8**), supporting the hypothesis that only weak interactions occur between ITZ and DCM. Moreover, **Fig. SI9** presents the comparison of FTIR spectra between the V-ITZ and SE-ITZ samples (SE-ITZ 20, 30, and 40) prepared from the ITZ: DCM mixtures after complete evaporation of solvent, and demonstrates no quantitative differences between the shape and position of their IR bands. This suggests that the resulting SE-ITZ structures retain similar features of the intermolecular interactions as the structure of V-ITZ.

In summary, the FTIR spectroscopy studies excluded the formation of any strong interactions between ITZ and DCM, which would somehow support the $\:Sm$ organization of ITZ molecules. It is consistent with the results of XRD studies presented in **Fig. ****1**, which demonstrate that in the freshly prepared ITZ:DCM mixture, there is no sign of $\:Sm$ order of ITZ. Therefore, the mechanism of $\:Sm$ order formation in ITZ may be sought in the process of the DCM evaporation itself without any significant bonding between ITZ and solvent. In fact, the process of the formation of an orientational order in a substance that was solved is known as evaporation-induced self-assembly (EISA) and has gained significant attention in recent years^32,33^. It may occur based on different mechanisms including the Marangoni flows, stick-and slip motion, and various deposition patterns near the contact line, giving rise to unique morphologies. Anisotropic materials, such as liquid crystalline compounds, tend to undergo self-assembly in solutions and the EISA is frequently utilized to assemble elongated particles mainly parallel to the contact line or along the evaporation flow. For example, when an aqueous solution of deoxyribonucleic acid (DNA) is evaporated on a substrate, the DNA chains extend linearly along the moving direction of the contact line due to the receding force^34^. Another example are ZnO nanorods – it was demonstrated that they may create a nematic alignment by a simple solvent evaporation technique and concluded that the degree of alignment varies with nanorod length, concentration and solvent evaporation rate^35^. High concentrations led to immediate aggregation of rods, less concentrated dispersions experienced stick–slip motion leading to thick stripes, while further decreasing the concentration resulted in thin parallel stripes oriented parallel to the evaporation line, and individual ZnO nanorods showed similar orientation. Moreover, it was demonstrated that high evaporation rates (> 4 μm/min) led to highly disordered thick stripes, whereas slow evaporation rates (< 2 μm/min) resulted in thin homogeneous stripes. We find an analogy in these outcomes to the ITZ:DCM systems, where for high ITZ concentrations in solvent the crystallization was observed, less concentrated dispersions led to the formation of the $\:Sm$ organization, while decreasing the concentration enabled the growth of the $\:Sm$ order. If the evaporation process is fast enough, the initial arrangement of molecules induced by the evaporation may be frozen. When a solution with a lower ITZ concentration is evaporated, the anisotropic ITZ molecules may be aligned due to the force induced by the evaporation flow, and, consequently, create a well-ordered structure that may be frozen in this state, as during the vitrification. However, with an increase in the ITZ concentration, there are fewer solvent molecules per each ITZ molecule, so their alignment may not be so effective, and the final frozen state after solvent evaporation may be characterized by a lower degree of the liquid-crystalline order. When it comes to evaporation rate, we found that very slow evaporation rates led to the ITZ crystallization, while faster rates resulted in the formation of the $\:Sm$ domains. In addition, it is known from the literature that the extremely fast evaporation rate used in the spray-drying method causes the formation of more disordered ITZ particles with $\:N$ alignment. Therefore, there is a certain dependence of the degree of structural order on the rate of solvent evaporation. Essentially, other literature reports have already pointed out that the rate of solvent evaporation is a crucial parameter for controlling the alignment of liquid crystals^29,36^. Since the IR studies did not indicate the formation of strong bonds between ITZ and DCM solvent, the hypothesis on the EISA-based mechanisms with a key role of the evaporation rate parameter seems quite plausible for the case of ITZ system. Moreover, it is worth mentioning the phenomenon of molecular recognition directed self-assembly, which is the ability of molecules to selectively bind to each other based on their shapes, chemical properties, and non-covalent interactions, resulting in the formation of ordered structures^37,38^. Such events between ITZ and the applied solvent molecules in their mixtures cannot be ruled out, especially in the context of possible polar interactions between ITZ and the solvents. Although ITZ is a weakly polar molecule, it is composed of nitrogen-containing polar rings, and both solvents employed in this work for dissolution of ITZ, DCM and CLF, are polar. Therefore, weak polar interactions between ITZ and DCM/CLF may be the case, while it was demonstrated in literature on molecular recognition phenomena that such interactions may cause a polar order and stabilize molecular packing in the $\:Sm$ structure^39,40^. Last but not least, molecular recognition events play an important role during the crystallization of different polymorphic forms from solutions^41–43^. For instance, a metastable form that persists in a solvent may be the consequence of specific solute − solvent interactions. In the case of ITZ, it was demonstrated that the $\:Sm$ structures obtained in the SE experiments are metastable and tend to crystallize to polymorphic form I. It cannot be ruled out that this is the result of the molecular recognition phenomena. However, a detailed mechanism of the formation of $\:Sm$ order in the solvent-evaporated itraconazole particles remains foggy. Understanding of this process and the role that the solvent plays requires considering the complex interplay between thermodynamic, kinetic, and molecular recognition factors.

## Conclusions

In this study, we characterized the structures of solid particles of a model thermotropic liquid crystal – itraconazole, obtained by a simple solvent evaporation method. We found that these particles exhibit smectic organization of molecules, which may be tuned by the concentration of itraconazole in the solvent. The character of the smectic order and the size of the ordered domains in itraconazole derived by its solvation in dichloromethane, followed by solvent evaporation, differ significantly from the smectic arrangement in the glassy state formed by supercooling of liquid. Namely, the applied solvent evaporation procedure results in reduced molecular offsets perpendicular to the layers as well as in much bigger smectic domains compared to those observed in itraconazole glass obtained by the vitrification. The lower the concentration of itraconazole in the mixture with solvent, the higher the degree of the smectic order quantified as sharpening and increase in the amplitude of the diffraction peaks and long-range pair distribution function oscillations. However, the physical stability (the resistance to crystallization) of itraconazole obtained from mixtures with dichloromethane of different solute to solvent concentrations turned out to be inversely proportional to the higher degree of the smectic order. The enhanced smectic order in powders received from the solvent evaporation method, as compared to that of glass obtained by vitrification, is metastable and tends to revert back towards a more disordered smectic structure with the storage time. Moreover, the crystallization occurs faster for the solvent-evaporated systems characterized by a higher degree of smectic organization. After heating the solvent-evaporated itraconazole systems to the liquid state, the smectic organization is disrupted, and their structure, as well as thermal properties and molecular dynamics, unify and tend to resemble those reported for glass obtained by vitrification.

Although the mechanism of the formation of smectic order in the solvent evaporation process has not been revealed, it was found that there are no strong interactions between the solvent and the itraconazole in their solutions. Understanding of this process and the role that the solvent plays requires considering the complex interplay between thermodynamic, kinetic, and molecular recognition factors. However, from the application point, it was demonstrated that the particles of itraconazole with the smectic structure obtained from the solvent evaporation method are characterized by higher release in 0.1 M HCl aqueous solution than commercially available crystalline powder. This knowledge is crucial for the development of liquid-crystalline systems with tailored structure and properties for applications in electronics and pharmaceutics.

## Supplementary Information

Below is the link to the electronic supplementary material.

Supplementary Material 1

## Data Availability

Research data supporting the results of the manuscript have been deposited in the RepOD repository: https://repod.icm.edu.pl/api/access/datafile/70081.
