# Supplementary material for: Effect of solvent evaporation on the liquid-crystalline order of itraconazole
Source: Sci Rep. 2025 Nov 17;15:40129. doi: 10.1038/s41598-025-23979-9 (PMC12623910; doi:10.1038/s41598-025-23979-9)
Supplement: Supplementary file 1 — Supplementary Material 1 [file 41598_2025_23979_MOESM1_ESM.docx]

**Supplementary Information**

**Effect of solvent evaporation on the liquid-crystalline order of itraconazole**

Taoufik Lamrani^1^*, Luiza Orszulak^2^, Magdalena Tarnacka^1^, Barbara Hachula^2^, Klaudia Nowakowska^3^, Kamil Kaminski^1^, Karolina Jurkiewicz^1^*

^1^ *Institute of Physics, Faculty of Science and Technology, University of Silesia in Katowice, 75 Pulku Piechoty 1, 41-500 Chorzow, Poland*

^2^ *Institute of Chemistry, Faculty of Science and Technology, University of Silesia in Katowice, Szkolna 9, 40-006 Katowice, Poland*

^3^ *Department of Biomedical Physics, Faculty of Physics and Astronomy, Adam Mickiewicz University in Poznan, Uniwersytetu Poznanskiego 2, 61-614 Poznan, Poland*

*corresponding authors: [taoufik.lamrani@us.edu.pl](mailto:taoufik.lamrani@us.edu.pl) (Tel. +48 32 349 76 24), [karolina.jurkiewicz@us.edu.pl](mailto:karolina.jurkiewicz@us.edu.pl) (Tel. +48 32 349 76 24)

**Studies of residual dichloromethane content
in solvent-evaporated itraconazole**


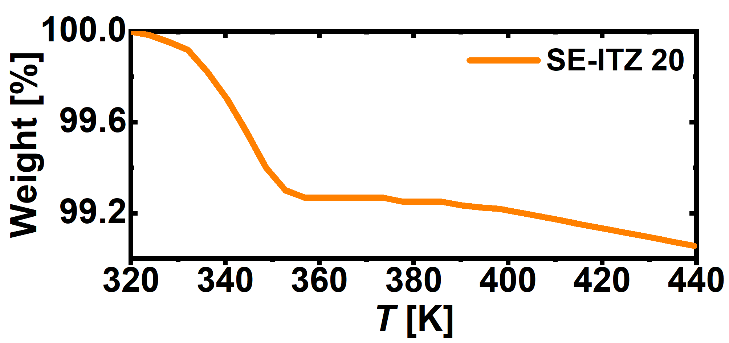


**Fig. SI1** Thermogravimetry curve of SE-ITZ 20 sample.

**Supporting X-ray scattering (diffraction) patterns
of solvent-evaporated itraconazole**


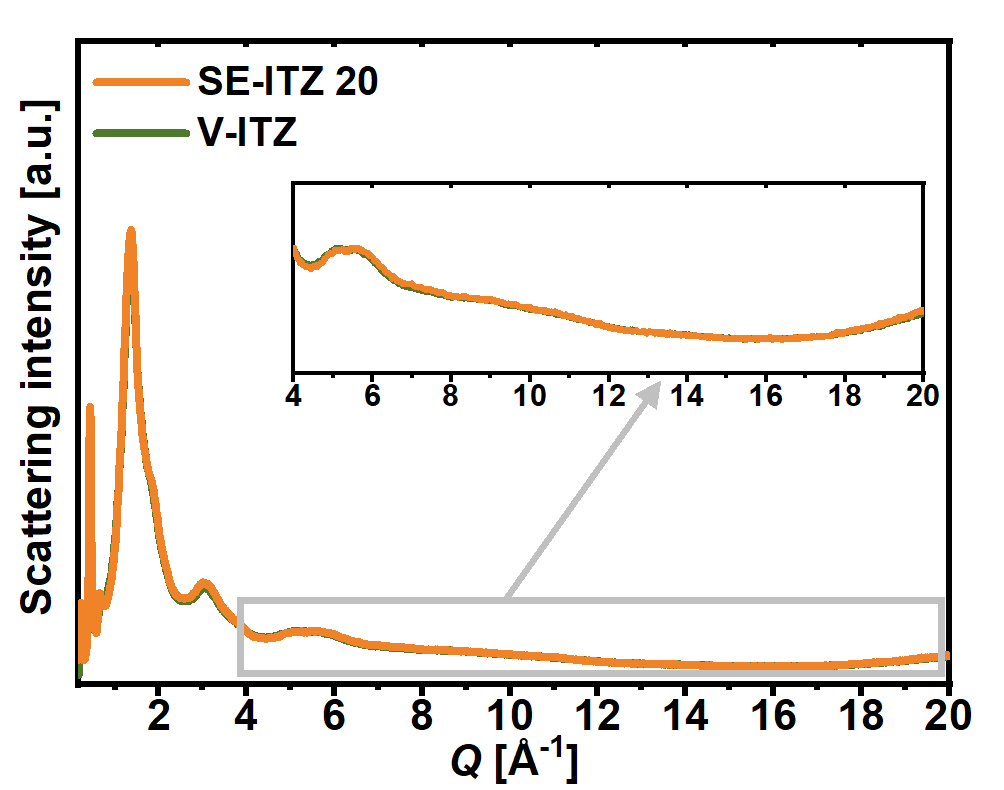


**Fig. SI2** X-ray scattering patterns of SE-ITZ 20 and V-ITZ in the full probed $Q$ range
of 0.17 – 20 Å^-1^, with insets highlighting the $Q$ region of 4 – 20 Å^-1^, used for intensity normalization.

**Fig. SI3** X-ray scattering pattern of SE-ITZ 40 stored at 295 K for 5 months.

**Fig. SI4** X-ray scattering patterns of fresh V-ITZ and SE-ITZ 30: fresh and stored at 313 K for 1, 3, 5, 7, and 14 hours. The inset presents the gradual decrease in the (002) diffraction peak’s amplitude for SE-ITZ 30 over the storage time.

**Drug release studies**

Before the drug release studies, the tested samples, SE-ITZ 40, V-ITZ and commercial crystalline ITZ, were pulverized in a mortar. The mixtures of each ITZ powder (30 mg) and 0.1M HCl in water (1 ml) were introduced into a dialysis cellulose membrane bag (MWCO 3.5 kDa), which was placed into a glass vial with 30 ml of 0.1M HCl solution and stirred at 310±0.2 K in an oil bath. The dialysis was carried out for 3 hours. The samples (100 μl) were taken from the release medium at appropriate time intervals (15, 30, 45, 60, 75, 90, 105, 120, 135, 150, 165, and 180 min) and dissolved in ethanol (1 ml) to determine the concentration of dissolved drug by UV–Vis spectroscopy method. Each experiment was repeated three times. All measurements were performed using Agilent Technologies Cary 60 UV-Vis spectrophotometer and Cary WinUV software for data processing.

ITZ solutions in ethanol of different concentrations were measured to determine the calibration curve (see **Fig. SI5** and **Fig. SI6**). Additionally, to confirm the adequate sensitivity of our UV-Vis apparatus for the determination of even low drug concentrations, the LOD (Limits of Detection) and LOQ (Limits of Quantification) parameters were calculated based on the constructed calibration curve using the formulas/equations below:

$$LOD=3.3\cdot\frac{\sigma}{S}$$

$$LOQ=10\cdot\frac{\sigma}{S}$$

where $\sigma-$ the standard deviation of the background signal or the response at low concentration (standard deviation of the blank sample) and $S-$the slope of the calibration curve obtained from the linear regression of absorbance *vs.* concentration (**Fig. SI6**).

**Fig. SI5** UV-Vis spectra for ITZ at different concentrations (in the range of 0.3–15 μg/mL; solutions in ethanol solvent).

**Fig. SI6** The calibration curve for ITZ in ethanol.

**Fig. SI7** Drug release profiles for various ITZ forms: crystalline, V-ITZ and SE-ITZ 40,
in 0.1M HCl.

**Infrared spectra of the itraconazole-dichloromethane mixture**

Firstly, we measured ATR-FTIR spectra of the commercial systems, ITZ and DCM, at 295 K and the assignment of particular molecular motions to the vibrational frequency of molecules is shown in **Fig. SI8**. The FTIR spectrum of V-ITZ showed a series of bands between 3200 and 2600 cm^−1^ assigned to the aromatic (3126 and 3066 cm^-1^) and aliphatic (2967, 2936, 2878, 2827, and 2764 cm^-1^) C-H stretching vibrations. The signal observed at 1697 cm^-1^ is related to the stretching vibrations of carbonyl groups. As reported in the literature [27,28] four peaks located at 1612, 1586, 1552, and 1509 cm^-1^ correspond to the aromatic C-C stretching, and the bands at 1450, 1379, and 1330 cm^-1^ are associated with the stretching vibrations of the N=N and C-N triazole moieties. The signals observed at 1271 and 1182 cm^-1^ are assigned to the aromatic C-H in-plane bending, while that at 1226 cm^-1^ can be connected with the asymmetric C-O-C stretching. The C-Cl stretching modes appear as a weak peak at 1106 cm^-1^. The asymmetric (cyclic ether) and symmetric (alkyl-aryl ether and cyclic ether) C-O-C stretching vibrations are visible at 1039 and 974 cm^-1^. The peaks occurring at 820, 794, 735, and 676 cm^-1^ can be assigned to the out-of-plane bending of the C-H benzene and triazole rings. On the other hand, in the DCM IR spectrum, two peaks detected at 3054 and 2987 cm^-1^ are assigned to the asymmetric and symmetric stretching of C-H bonds, respectively. The signals located at 1422 and 1264 cm^-1^ correspond to the bending vibrations of C-H groups, whereas the doublet visible at 732 and 703 cm^-1^ is related to the asymmetric and symmetric stretching of C-Cl groups.

Next, FTIR spectra of the ITZ:DCM mixtures were recorded to obtain more information about the interactions between solute and solvent molecules and are illustrated in **Fig. SI8**.

**Fig. SI8** FTIR spectra of V-ITZ and DCM as well as ITZ:DCM mixtures for 20, 30, and 40 mg/ml concentrations. Data were presented in two spectral regions: (left) 3200–2600 cm^−1^ and (right) 1800–400 cm^−1^.

Additionally, a comparison of the IR spectra of V-ITZ and the SE-ITZ samples prepared from ITZ:DCM mixtures of different concentrations (20, 30, and 40 mg/ml) is presented in **Fig. SI9.**


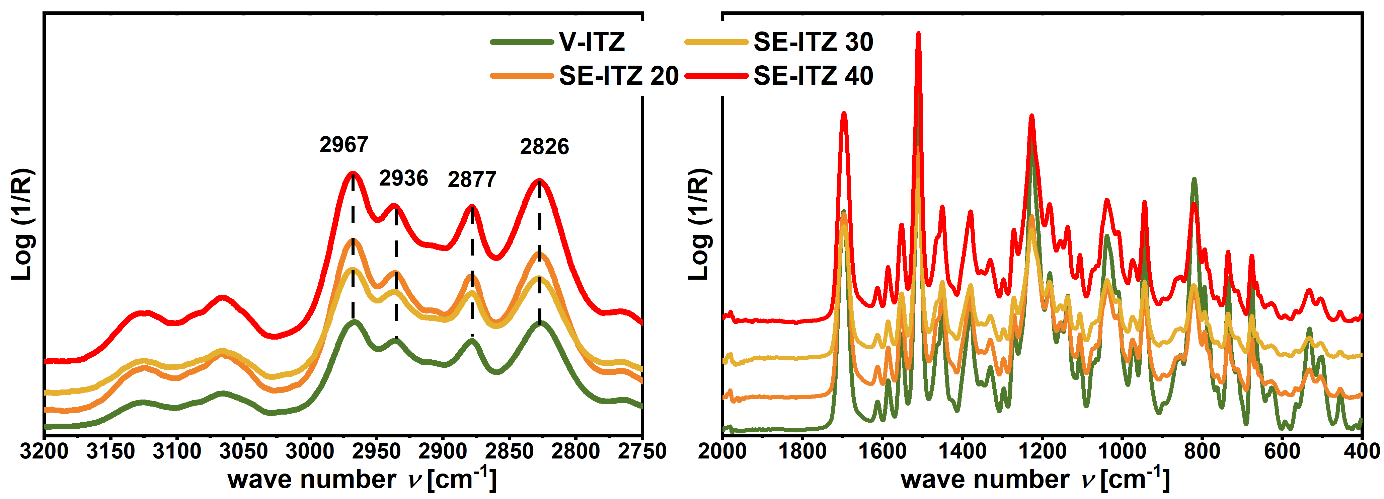


**Fig. SI9** FTIR spectra of SE-ITZ samples, prepared from ITZ:DCM mixtures of different concentrations (20, 30, and 40 mg/ml), compared with that of V-ITZ, presented in two spectral regions: (left) 3200–2750 cm^−1^ and (right) 2000–400 cm^−1^.
